# Sex difference in the weighting of expected uncertainty under chronic stress

**DOI:** 10.1038/s41598-021-88155-1

**Published:** 2021-04-22

**Authors:** Huijie Lei, Yasuhiro Mochizuki, Chong Chen, Kosuke Hagiwara, Masako Hirotsu, Toshio Matsubara, Shin Nakagawa

**Affiliations:** 1 Division of Neuropsychiatry, Department of Neuroscience, Yamaguchi University Graduate School of Medicine, Ube, Yamaguchi Japan; 2 RIKEN Center for Brain Science, Wako, Saitama Japan

**Keywords:** Human behaviour, Computational neuroscience, Decision, Stress and resilience

## Abstract

The neurobiological literature implicates chronic stress induced decision-making deficits as a major contributor to depression and anxiety. Given that females are twice as likely to suffer from these disorders, we hypothesized the existence of sex difference in the effects of chronic stress on decision-making. Here employing a decision-making paradigm that relies on reinforcement learning of probabilistic predictive relationships, we show female volunteers with a high level of perceived stress in the past month are more likely to make suboptimal choices than males. Computational characterizations of this sex difference suggest that while under high stress, females and males differ in their weighting but not learning of the expected uncertainty in the predictive relationships. These findings provide a mechanistic account of the sex difference in decision-making under chronic stress and may have important implications for the epidemiology of sex difference in depression and anxiety.

## Introduction

An important insight from recent studies in the field of decision-making is that psychological stress affects decision-making, but with sex differences^1,2^. Specifically, in the face of acute stress, males and females seem to show distinct patterns in their perception of probabilistic uncertainty (or risk preferences). Males tend to become more risk-seeking while females more risk-aversive, as demonstrated in experimental tasks with explicit probabilistic information such as the Game of Dice Task^3^ and the Cambridge Gambling Task^4^. Similar results have been reported in ambiguous situations where learning of the cue-outcome contingencies (i.e., reinforcement learning, RL) is required, such as in the Iowa Gambling Task^5,6^ (where risk-taking is financially disadvantageous) and the Balloon Analogue Risk Task^7^ (where risk-taking is advantageous).

However, despite these fruitful findings with acute stress, little is known about the effects of chronic stress on decision-making. Unlike acute stress which often induces fast, adaptive responses that enhance survival, chronic stress causes prolonged, maladaptive responses that contribute to neuropsychiatric disorders in particular depressive and anxiety disorders (hereinafter, depression and anxiety)^8,9^. Recent evidence from the neurobiological literature suggests that chronic stress alters reward and threat processing in the brain (i.e., decision-making), which ultimately leads to the development of depression and anxiety^10–12^. Whereas a striking sex difference has been consistently identified in the prevalence of depression and anxiety (i.e., females are twice as likely to be affected)^13–15^, whether the effects of chronic stress on decision-making is sex-dependent remains unclear. Clarifying the potential sex difference in the effects of chronic stress on decision-making may shed light on the epidemiology of sex in depression and anxiety and provide insights into the psychopathology as well as prevention and treatment of these disorders.

In the present study, we set out to investigate the effects of chronic stress on decision-making in healthy human subjects and sought to identify potential sex differences in these effects. For such purpose, we employed a RL based decision-making paradigm. Given that the outcomes of our decisions in everyday life are often uncertain, the ability to use effective cues to predict the outcomes is essential. RL or trial-and-error learning of such predictive relationships (i.e., cue-outcome contingencies) constitutes a fundamental form of human learning from direct interaction with the environment^16^. In fact, RL has been considered a key decision-making mechanism involved in the development and treatment of depression and anxiety. For instance, relearning of emotional associations in social interactions via adequate RL has been considered a prerequisite for the therapeutic effect of antidepressants^17^. Meanwhile, altered RL of aversion information plays a key role in the development of anxiety and has been considered a primary treatment target^18,19^.

Importantly, RL provides a computational framework for encapsulating the mechanistic processes involved in associative learning as well as learning-based decision-making. RL-based decision-making involves two independent computational processes, an initial RL of the probabilistic cue-outcome contingencies followed by a subsequent weighting of the learned probability or uncertainty. This uncertainty is known as “expected uncertainty” or “irreducible uncertainty” because the uncertainty or unreliability of predictive relationships is expected and not reduced by gathering more experience^20–22^. A related concept is “risk”, where the uncertainty is expressed as explicit probabilities and not to be learned. Recent research suggests that learning and weighting of expected uncertainty involve different cognitive and neural computations in the brain^20,23^ and successful decision-making requires the proper implementation of both of them.

We predicted that males and females would perform differently in RL-based decision-making under chronic stress. Furthermore, the potential sex difference in RL-based decision-making might be explained by a sex-specific change in either learning or weighting of the expected uncertainty, or a combination of both. Therefore, in contrast to the current RL literature on depression and anxiety^24–26^ that has primarily focused on quantifying the change of learning, we used a RL task and computational models^27,28^ that allowed us to tease apart the influence of the learning component (i.e., learning rate) and the weighting component involved in the decision-making process.

## Results

### Females choose “correct” options less often than males, but only under high stress

**Figure 1:**
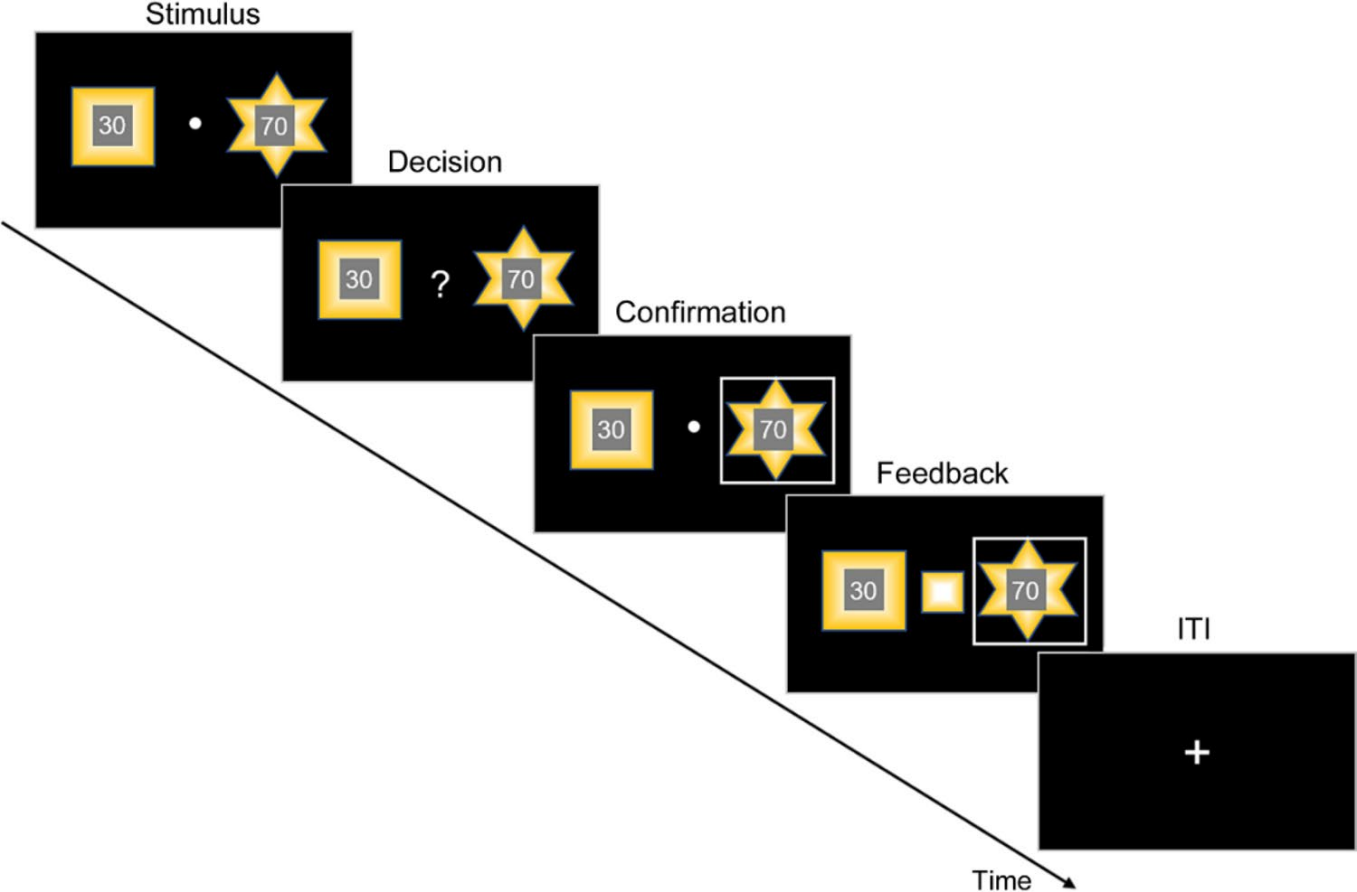
Illustration of the RL-based decision-making task. Each trial consisted of four stages, STIMULUS, DECISION, CONFIRMATION, and FEEDBACK. Two fractal stimuli each with a number in their center were shown to participants (STIMULUS phase). Participants were told that the fractal stimulus indicated reward probability which they had to learn through trial-and-error experience and that the number shown in the center represented the reward magnitude which they would receive once the stimulus was rewarded. Participants had three seconds to indicate their choice by pressing one of two arrow keys and were instructed to respond as soon as possible once they had made the decision (DECISION phase). The chosen stimulus was immediately highlighted by a gray frame (CONFIRMATION phase). Later, the rewarded stimulus was revealed in the center (FEEDBACK phase). ITI, inter-trial interval.

Subjects performed a RL-based decision-making task (Fig. 1), choosing between two fractal stimuli based on their reward magnitude (shown in the center) and reward probability. One of the two fractal stimuli was arbitrarily assigned a higher reward probability (0.75 versus 0.25), and subjects had to learn this probability assignment through trial-and-error experience. This task design with a fixed, probabilistic stimulus-outcome association allowed us to evaluate the learning and weighting of expected uncertainty. The proportion of choosing the option/stimulus with high expected value (i.e., “correct” choices) among all trials as well as in each block was calculated for each participant and used as the primary model-free measure of the task performance. Expected value was calculated as the product of the true reward probability (0.75 versus 0.25) and the magnitude of each option.

We asked participants to indicate their perceived psychological stress in the past month using the widely employed Perceived Stress Scale (PSS)^29,30^. Participants’ mean ± SD score on this scale was 18.71 ± 5.37, comparable to a previous report with young adults of similar background^30^. We categorized participants (n = 65; see Supplementary Table S1 for demographic information) as perceiving low versus high chronic stress based on a mean split of their score on the scale. Consequently, the mean ± SD score of PSS for each group was 14.89 ± 3.06 and 23.17 ± 3.79, respectively. We considered this dichotomization meaningful since it has been generally suggested that a score of 20 or higher on PSS represents a high level of stress^31,32^.

**Figure 2:**
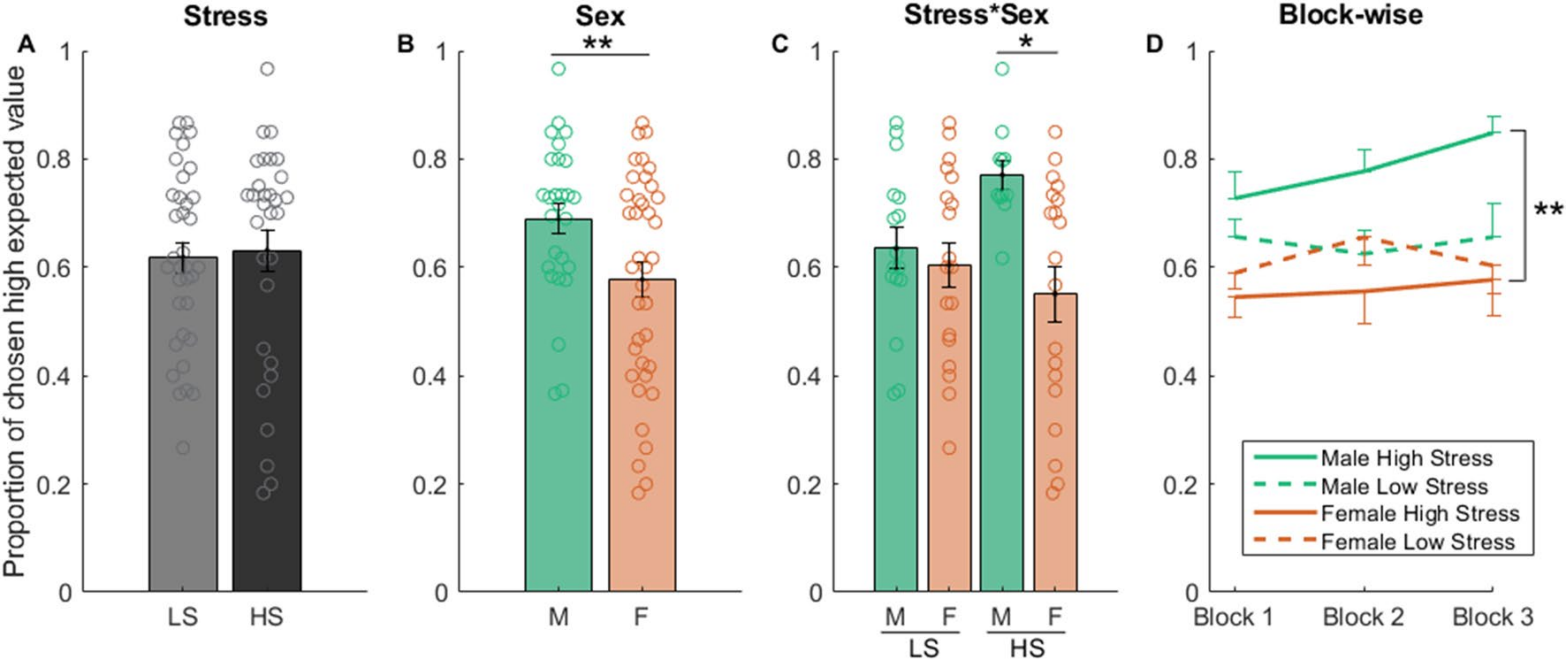
The effect of stress and sex on task performance. The effect of stress (**A**), sex (**B**), and stress*sex interaction (**C**) on the proportion of choosing the option with high expected value (“correct” choices). Block-wise analysis of the data is shown in (**D**). For (**A**)–(**C**), each circle represents a subject. Error bars represent SEM. *p < 0.05, **p < 0.01, two-tailed. *LS* low stress, *HS* high stress, *M* male, *F* female.

As shown in Fig. 2, a stress and sex two-way ANOVA test indicated a significant effect of sex (*F*_1,61_ = 7.990, p = 0.006) and sex*stress interaction (*F*_1,61_ = 4.603, p = 0.036) but not stress (*F*_1,61_ = 0.849, p = 0.360) on the proportion of choosing the option with high expected value (Fig. 2A–C). Post hoc tests revealed that under high stress but not low stress, females chose the option with high expected value less frequently than males (p = 0.004 for high stress, p = 0.594 for low stress; Fig. 2C). As can be seen, under high stress, females chose about 20% less correct options compared to males. This tendency was confirmed across the three blocks of trials (repeated measures ANOVA: *F*_1,28_ = 10.322, p < 0.01; Fig. 2D). A further within-sex analysis suggested, however, that the sex difference under high stress was primarily driven by enhanced performance in males (high versus low stress within males, for overall performance: *F*_1,25_ = 7.069, p = 0.013; for block-wise performance: *F*_1,25_ = 7.272, p = 0.012) rather than impaired performance in females (high versus low stress within females, for overall performance: *F*_1,25_ = 0.705, p = 0.407; for block-wise performance: *F*_1,25_ = 0.775, p = 0.385).

### Computational model-based analysis: different weighting of expected uncertainty explains the sex difference in choice performance under high stress

To quantitatively capture the computational process underlying RL, we fitted eight computational models to participants’ choice behaviors. These included two static RL models, two dynamic RL models as well as their variates incorporating a probability weighting parameter (cf. “Methods”for details).

To fit the above models to each participant’s behavior, we employed *maximum *a posteriori (MAP) estimation, a Bayesian-based approach that incorporates prior belief about parameter values to avoid overfitting common in a maximum likelihood approach^33^. The mean model evidence for each model across all subjects is presented in Table 1. As can be seen, the four original models all underperformed their variates that included a probability weighting parameter. It suggests that participants do not simply use predicted probability in their decision-making but weight that probability in a subjective way. Importantly, the winning model was model s1w that had a single learning rate and a probability weighting parameter (Table 1).

**Table 1:** Model specification and fitting results.

Model description		Model name	Free parameters	Mean model evidence
No probability weighting parameter	Static	s1	α, β	− 35.8708
		s2	α_+_, α_−_, β	− 32.8888
	Dynamic	d1	μ, κ, β	− 45.4718
		d2	α_1_, α_2_, β	− 35.6651
Probability weighting parameter	Static	**s1w**	**α**, **γ**, **β**	− **32.2318**
		s2w	α_+_, α_−_, γ, β	− 32.6815
	Dynamic	d1w	μ, κ, γ, β	− 40.4454
		d2w	α_1_, α_2_, γ, β	− 32.6518

The winning model is shown in bold.

To further evaluate whether the winning model actually captured participants’ behaviors, following Boorman et al., 2011^34^, we conducted a generalized linear mixed model analysis to determine the degree to which choosing one option was predicted by both the reward magnitude and the reward probability as estimated by the best model s1w. Results indicated a significant positive effect of both reward magnitude and the model estimated reward probability in all subjects as well as in subjects of each sex-stress group (p < 0.001; Supplementary Fig. S1).

**Figure 3:**
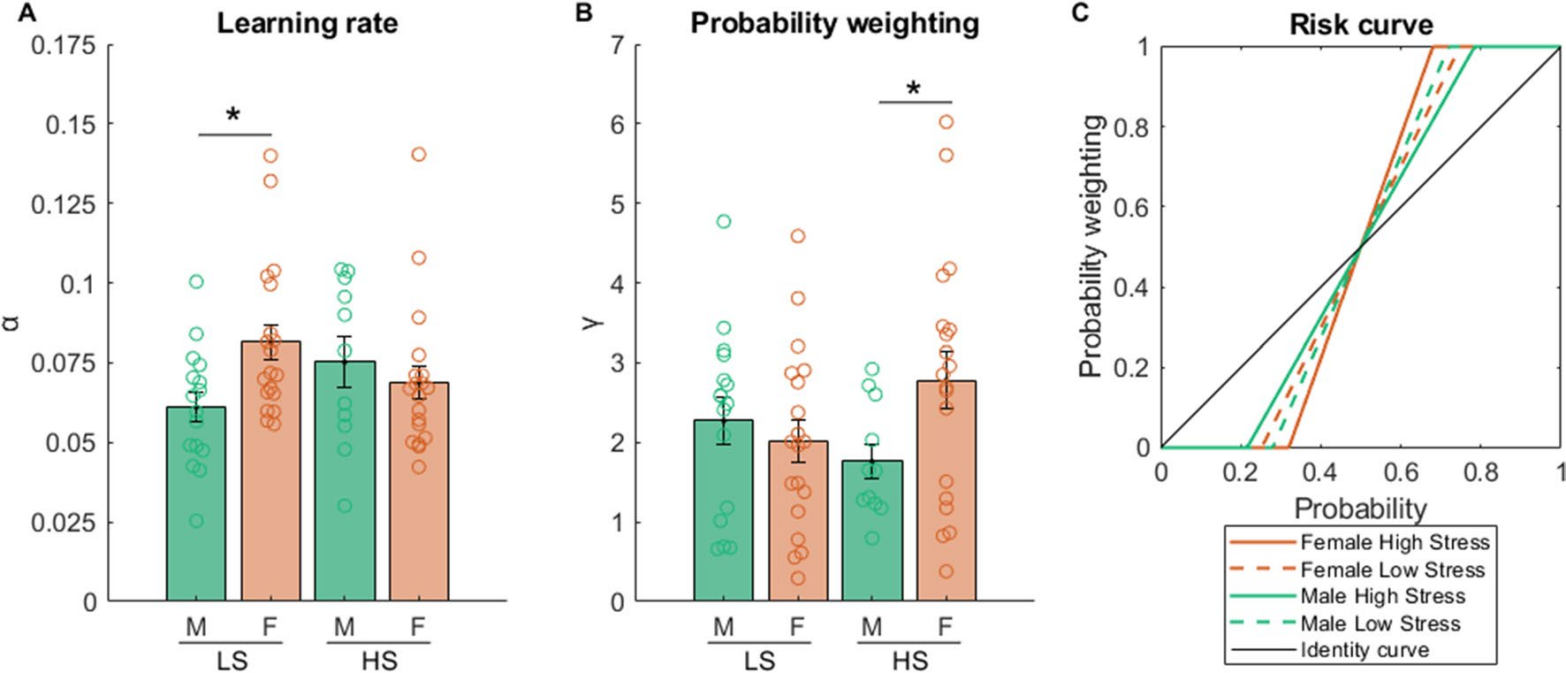
Computational model-estimated learning rate and probability weighting parameter across sex and stress groups. (**A**), learning rate. (**B**), probability weighting. To illustrate this sex difference, a representative plot of the risk curve for each sex-stress group using the group mean of the probability weighting parameter is shown (**C**). *p < 0.05. *LS* low stress, *HS* high stress, *M* male, *F* female.

The scatterplot of the estimated learning rate and probability weighting for all participants in each stress-sex group is shown in Fig. 3A,B. A stress and sex two-way ANOVA test indicated a significant stress*sex interaction for both learning rate (*F*_1,61_ = 5.293, p = 0.025) and probability weighting (*F*_1,61_ = 4.168, p = 0.046). For learning rate, post hoc tests revealed a significant sex difference under low stress (*F*_1,33_ = 7.605, p = 0.009), without any other differences. Thus, under low stress, females learned faster than males. For probability weighting, post hoc tests revealed a significant sex difference under high stress (*F*_1,28_ = 4.218, p = 0.049) and a trend towards significance for low versus high stress within females (*F*_1,36_ = 2.990, p = 0.092), without other differences. Thus, under high stress, females underweighted small probabilities and overweighted large probabilities to a greater degree compared to males. In other words, females showed greater risk aversion than males. This sex difference in probability weighting under high stress seems to be the results of an increase in probability weighting with a trend towards significance in females and a nonsignificant decrease in probability weighting in males due to stress. A representative plot of this sex difference using the group mean is shown in Fig. 3C.

**Figure 4:**
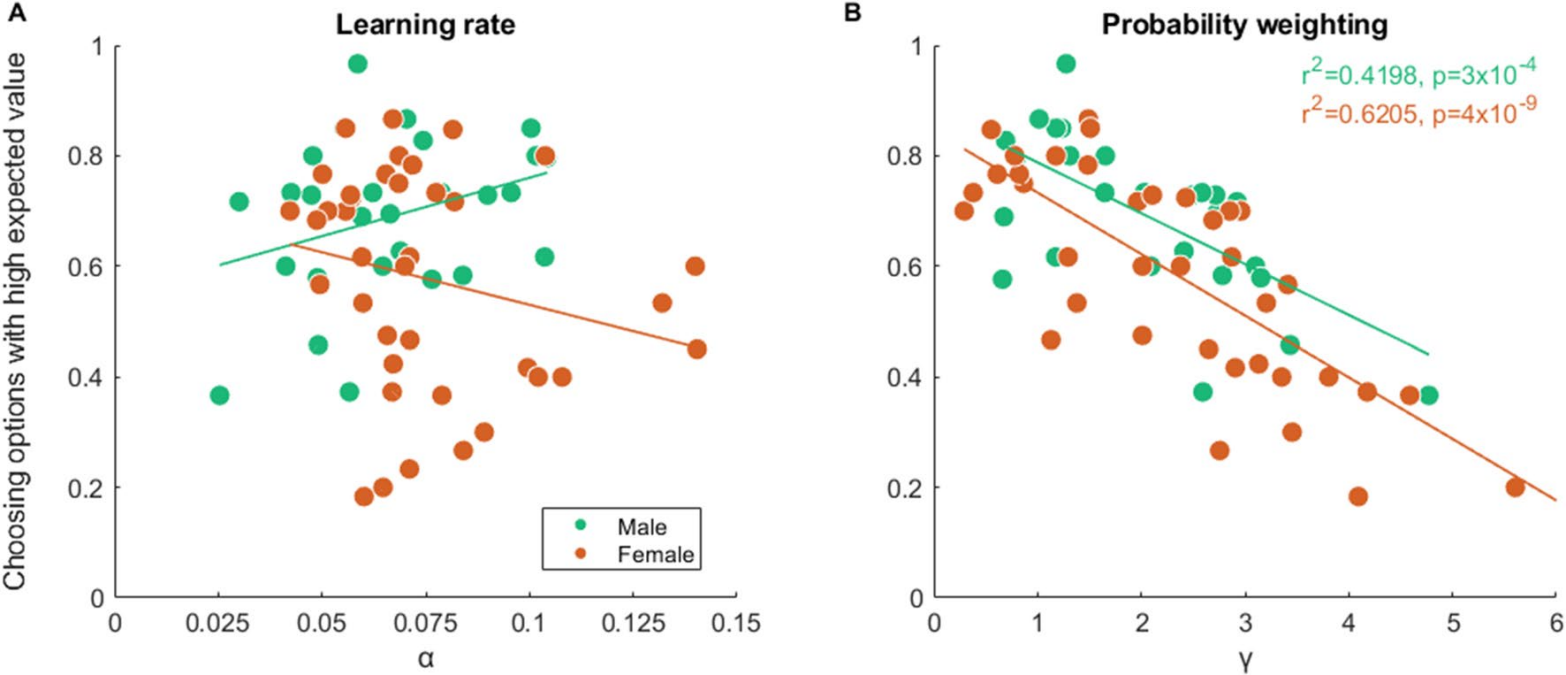
Associations between task performance and model estimated parameters. Scatterplot (with regression lines) of the proportion of choosing the option with high expected value as a function of learning rate (**A**) and probability weighting (**B**) in each sex, respectively. Subjects of low and high stress were combined for each sex group. Green indicates males and orange females.

Meanwhile, we found that greater probability weighting (linear regression, r^2^ = 0.4180 for males, r^2^ = 0.6188 for females, both p < 0.001) but not learning rate was associated with a lower proportion of choosing correct options in both sexes (Fig. 4). Similar associations were obtained for block-wise analysis (Supplementary Fig. S2).

**Figure 5:**
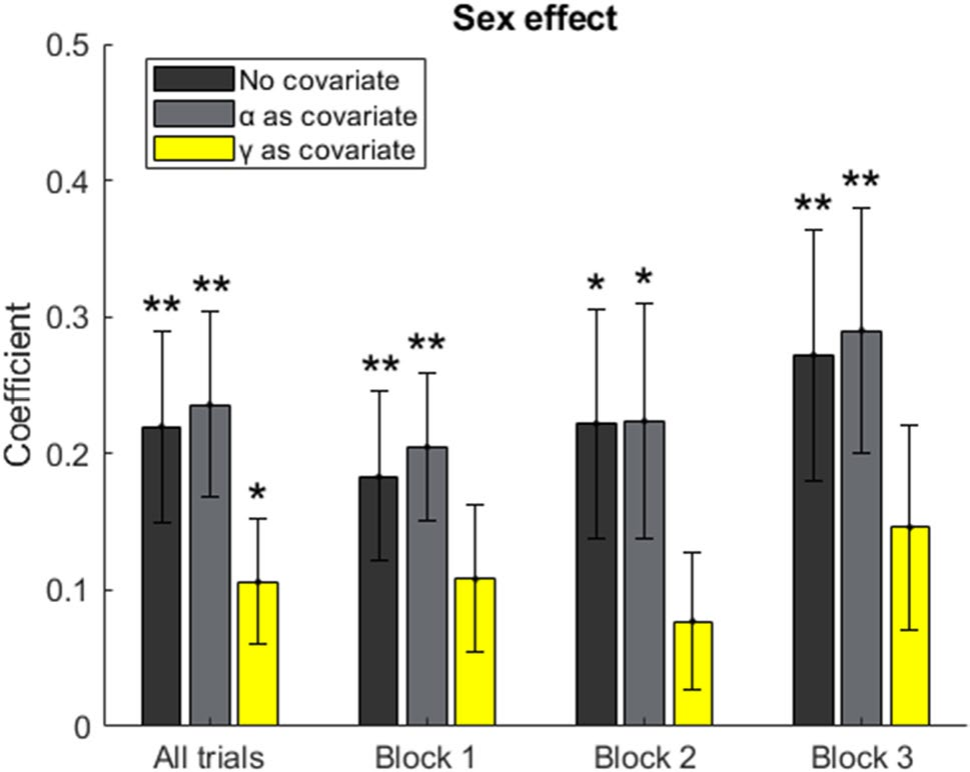
Parameter estimate for the sex difference in task performance under high stress. The coefficients (unstandardized) are obtained using general linear models with the proportion of choosing the option with high expected value as dependent variable and sex as the independent variable (male = 1, female = 0). Error bars represent SEM. *p < 0.05, **p < 0.01.

We further tested whether the sex difference in choosing correct options would disappear or be greatly attenuated by statistically controlling the influence of the parameters we estimated. For such purpose, we first quantified the sex difference by fitting a general linear model, with the proportion of choosing correct options as the dependent variable and sex as the independent variable (male = 1, female = 0). The unstandardized regression coefficient was 0.220 ± 0.070 (mean ± SE) among all trials (p < 0.01, Fig. 5, dark gray, no covariate, all trials), indicating that under high stress, females chose 22% less correct options compared to males. The parameter estimate for this sex difference remained stable across the three blocks (Fig. 5, dark gray, no covariate, block 1 ~ 3). We next incorporated the estimated parameters as the covariate and found that after controlling the probability weighting parameter γ but not learning rate α (Fig. 5, yellow versus light gray), the parameter estimate for the sex difference in choosing correct choices under high stress among all trials was greatly attenuated while that for each block became nonsignificant. This indicates that it is probability weighting rather than learning rate that drives the sex difference in task performance under high stress.

Finally, since it has been reported that RL may be associated with cognitive capacities^35^ and that chronic stress affects cognitive capacities^36^, we also administered a n-back task to examine the potential involvement of working memory. We found that there was no effect of sex, stress, or sex*stress interaction on working memory, and working memory did not account for the sex difference in choosing correct choices under high stress (Supplementary Fig. S3).

## Discussion

In the present study, we found a sex difference in choice performance under high stress in a sample of young adults: females choose about 20% less correct options compared to males. This sex difference is primarily driven by enhanced performance in males rather than impaired performance in females by chronic stress. In contrast, we did not find a sex difference in choice performance under low stress. Applying computational models to dissect the underlying processes of RL-based decision-making, we found that in response to chronic stress, whereas males show a nonsignificant decrease in probability weighting, females show an increase in probability weighting with a trend towards significance. As a result, females show a greater probability weighting parameter than males such that they underweight small probabilities and overweight large probabilities to a greater degree compared to males. More importantly, the sex difference in choice performance is explained by different weighting rather than learning of expected uncertainty between sexes under high stress. To our knowledge, this is one of the first studies to examine the association between chronic stress and decision-making and the first study looking at sex differences in RL-based decision-making under chronic stress.

Thus, rather than the initial RL of the probabilistic cue-outcome contingencies, chronically stressed males and females differ in their weighting of the learned probability or uncertainty. Our results are partially consistent with one previous study of decision-making with explicit probabilistic information^37^. Kandasamy et al., 2014 administered hydrocortisone (pharmaceutical cortisol) to human volunteers over eight days to mimic a sub-chronic stress episode and found that males overweighted small probabilities while underweighted large probabilities relative to females^37^. In other words, males behaved in a more risk-seeking (i.e., less risk-aversive) way compared to females. Similar sex difference in risk preference has been reported in decision-making under acute stress^1,2^. In contrast to the psychiatric literature on RL^24–26^ that has primarily focused on the role of learning, here we show rather than learning per se, it is the distinct weighting of the expected uncertainty that drives the sex difference in decision-making under chronic stress. Specifically, under high stress, females are more risk-aversive than males. They underweight small probabilities and overweight large probabilities to a greater degree compared to males.

Previous research has also indicated that the sex difference in decision-making may be context-dependent. For instance, Li et al. showed that males and females demonstrate different changes in loss aversion when mating or self-protection motives are activated^38^. Specifically, when mating motives are induced, males tend to be less loss aversive or more gain seeking while females show no change or even a slight increase in loss aversion. In contrast, when self-protection motives are induced (with a fearful or stressful situation), males show a large increase in loss aversion while females do not show any change in loss aversion^38^. Since we did not examine this kind of context-dependent sex differences, future studies may investigate, for instance, whether there exists an interaction between chronic and acute stress in the sex differences in decision-making.

While we identified the existence of such sex difference in probability weighting under high stress, the biological and psychological explanations remain unclear. Previous research of sex difference in risk-seeking behaviors under explicit risk conditions has focused on the role of testosterone^39,40^. A lack of sex difference in risk aversion at low stress condition in the present study, however, suggests the possibility that testosterone and stress hormones may jointly affect risk preference in ambiguous decision-making that requires RL.

On the other hand, to date the literature on the psychopathological explanation of the sex difference in depression and anxiety shows that females ruminate (i.e., overthink about one’s negative emotional experience) more frequently^41,42^ and worry to a greater degree^43^ than males. Both rumination^44,45^ and worry^46^ are associated with more enhanced risk-aversive behaviors. Thus, the sex difference in probability weighting (or risk aversion) under high stress we observed might be due to sex difference in rumination and worry. In fact, more frequent rumination in females has been believed to largely explain the striking sex difference in the prevalence of depression (i.e., females are twice more likely to be affected by depression)^41,42^. Therefore, future research may investigate whether the different weighting of expected uncertainty provides a mechanistic explanation to the sex difference in rumination/worry and even the sex difference in the prevalence of depression and anxiety. Testing such a possibility may also propel us towards a better understanding of the high comorbidity of depression and anxiety^47,48^ since chronic stress is a common risk factor for both disorders.

Our observed sex difference might provide a potential explanation for why previous studies of patients with depression have failed to identify consistent deficits in RL of cue-outcome contingencies in static environments (i.e., fixed cue-outcome associations)^24,49,50^. In fact, no previous studies have ever investigated potential sex differences in RL under depression or anxiety. Neither did they separate the component of weighting from learning of the expected uncertainty. Based on our results, it is possible that perhaps depressed females might be more likely to perform suboptimally on RL-based decision-making than males, and this is to a large extent due to altered weighting rather than learning of expected uncertainty: females tend to underweight small probabilities and overweight large probabilities to a greater degree compared to males. Future research with depressed patients may test these possibilities using a similar computational framework.

Taken together, utilizing the computational approach, the present study provides a mechanistic account of the sex difference in RL-based decision-making under high stress. It provides an example illustrating how computational psychiatry may help bridge the explanatory gap between observable behaviors and the underlying specific cognitive computations in the brain^24^.

Our study also has several limitations. Firstly, the cross-sectional nature of the dataset did not allow us to draw causal conclusions. Secondly, only young subjects were investigated in this study. Future research should include subjects from all ages and in particular adolescents and older adults in whom the sex difference in mental health may be more emphasized^13^. Thirdly, although we observed a sex difference in probability weighting under high stress, our study had insufficient power to specify if the sex difference was due to a decrease in males or an increase in females due to stress, or both. Future studies are required to clarify the mechanism of the sex difference we observed here with larger sample sizes. Lastly, as we have mentioned, the uncertainty we investigated here is known as expected uncertainty or irreducible uncertainty. There is also another kind of uncertainty called “unexpected uncertainty” which involves changing cue-outcome associations^20–22^. It remains for future work to investigate whether sex difference exists in the computation of uncertainty in this kind of sophisticated, volatile environments.

## Materials and methods

### Participants

This research was part of an ongoing larger prospective study designed to predict mental health of young adults using evaluations of high-level cognitive functions. Data collected at the baseline of the study during the year 2019 were used for the analysis here. The study was carried out in accordance with the latest version of the Declaration of Helsinki and approved by the Institutional Review Board of Yamaguchi University Hospital. The inclusion criteria were being 20–39 years old at the time of the visit and the exclusion criteria were (1) having any self-reported psychiatric disorders, (2) receiving medical examinations due to suspicion of any psychiatric disorders, (3) being suspected of psychiatric disorders by the research staff and subsequently diagnosed as having any psychiatric disorder by the Mini-International Neuropsychiatric Interview conducted by a psychiatrist, or (4) being unable to perform the laboratory tests and answer the questionnaires for this study due to severe physical conditions or other reasons. No participant was excluded owing to meeting any of the exclusion criteria.

Among 68 participants that agreed to participate in this study and provided written informed consent after receiving a detailed explanation, 3 were excluded from the present analysis due to too many no response trials (over 8%) or data recording problems in the RL task, leaving 65 participants (mean age: 22.46 ± 5.91; range 20–38 years) for the final analyses. We categorized subjects as perceiving low versus high chronic stress based on a mean split of their score on PSS such that subjects scoring 19 and above were categorized as perceiving high stress (mean = 18.7). There were no stress-sex group differences regarding age or any other demographic information (see S1 Table S1).

### RL task

We used an established RL task design^27,28^. The task had sixty-trials and was programmed using MATLAB R2018b (MathWorks) and Psychtoolbox 3 (http://psychtoolbox.org/). In each trial (Fig. 1), participants were asked to choose between two fractal stimuli to maximize the number of reward points earned. Each stimulus was randomly assigned a reward magnitude shown in the center. In our experiment, we used complex fractals generated from the Mandelbrot set. Participants were told that the fractal stimulus indicated reward probability which they had to learn through trial-and-error experience and that on each trial, one of the stimuli would be rewarded. The two fractal stimuli were randomly positioned left or right for each trial. One of the two fractal stimuli was arbitrarily assigned a higher reward probability (0.75 versus 0.25). To ensure each participant experience the same stimulus-reward contingency (i.e., 0.75 versus 0.25), we randomized the reward assignment every twenty trials, resulting in three blocks. The reward magnitude for one stimulus (R) was randomly sampled from a uniform distribution ranging from 1 to 99, while that for the other stimulus was set to 100-R. Following Suzuki et al., 2012^28^, we added an adjustment to the reward magnitude to balance participants’ choices (i.e., the magnitude assigned to a stimulus was reduced once that stimulus had been chosen twice in a row). Subjects were also informed that the reward magnitude was independent of the probability.

The stimuli were shown for three seconds (STIMULUS phase), after which a question mark occurred and participants were instructed to indicate their choice by pressing one of two arrow keys within three seconds and as soon as possible once they decided their choice (DECISION phase). After making a response, the chosen option was highlighted by a gray frame (CONFIRMATION phase), and then the rewarded stimulus on that trial was shown in the center (FEEDBACK phase). Failing to press a key within the DECISION phase would be counted as no response and participants would not be able to earn any reward points on that trial. After excluding two participants who failed to respond on over 8% trials and one participant with data recording problems, the remaining sixty-five subjects had a no response rate of merely 0.34 ± 0.64% (mean ± SD).

In this study, each subject received a fixed payment of approximately 3000 JPY for participating in this study. We did not implement the performance-adjusted payment, because our final objective was to develop useful tools for predicting mental health problems in public health settings: it is impossible to pay people a certain amount of money based on their choices in any public health predictive tools. Meanwhile, previous research has shown that people’s decision-making with hypothetic rewards highly resembles that with real rewards^51–53^.

### Working memory task

The n-back task was programmed by Jörn Alexander Quent^54^ after Jaeggi et al., 2010^55^ using MATLAB and Psychtoolbox 3. Following the signal detection theory, a discriminability score (d_2_) indicating the overall performance at discriminating targets from non-targets^56^ was calculated for the 2-back task for each subject and used as an indicator of working memory.

### Data analysis

Data analysis was conducted with IBM SPSS Statistics 26 and MATLAB R2018b. All statistical tests were two-sided. We used two-way ANOVA to test the effects of stress and sex on choice behaviors and model parameters. We used general linear models to test the association between model parameter and choice behaviors and to quantify the observed sex differences.

### Computational modeling

To quantitatively capture the computational process underlying RL, we fitted eight computational models to participants’ choice behaviors (Table 1). These included two static RL models, two dynamic RL models as well as their variates incorporating a probability weighting parameter.

#### Static model 1 (s1)

In this model, after choosing stimulus A on trial *t* and observing reward *r*_*t*_ (1 if stimulus A is rewarded and 0 otherwise), the predicted probability for stimulus A is updated according to a standard Rescorla–Wagner model with a constant learning rate^16^:

1 $$p_{t + 1} \left( {\text{A}} \right) = p_{t} \left( {\text{A}} \right) + \alpha \cdot \delta_{t}$$

2 $$\delta_{t} = r_{t} - p_{t} ({\text{A}})$$

where α is the learning rate and *δ*_*t*_ is the probability prediction error. The predicted probability for stimulus B is modeled as *p*_*t*_(B) = 1 − *p*_*t*_(A). In our implementation, the predicted probability for each stimulus is initialized to 0.5. Then for each trial, the expected value of a stimulus Q_*t*_(·) is computed as the product of the reward magnitude and predicted probability of that stimulus *p*_*t*_(·).

#### Static model 2 (s2)

This model is identical to model s1 except that it uses two learning rates, α_+_ for positive and α_-_ for negative prediction errors. This model is incorporated because it has been frequently proposed that people may respond differently to positive versus negative feedback.

#### Dynamic model 1 (d1)

This model is known as the Pearce–Hall learning model^57^ which substitutes associability-gated dynamic learning rate for the constant learning rate in model s1. Unlike model s1, the learning rate in this model changes adaptively in every trial depending on the reliability of prior predictions (i.e., the associability S):

3 $$\alpha_{t + 1} = \kappa \cdot S_{t + 1}$$

4 $$S_{t + 1} = \left( {1 - \mu } \right) \cdot S_{t} + \mu \cdot \left| {\delta_{t} } \right|$$

where *κ* is the scale of learning rate, *S*_*t*+*1*_ is the associability on trial *t* + *1*, and *μ* is the step size for updating associability which determines the relative weight of the associability and the absolute value of prediction error on the previous trial *t*.

#### Dynamic model 2 (d2)

This simplified dynamic model is identical to model s1 except that it uses two learning rates, one for the first half and the other for the latter half of trials. This model was included to simulate the strategy of fast acquisition at first and subsequent stable choice^33^.

Each model described above is then combined with a probability weighting parameter γ that transforms the influence of predicted probability to account for risk-aversive or risk-seeking behaviors^27,28^:

5 $$F(p_{t} \left( \cdot \right),\gamma ) = \max \left[ {\min \left[ {\left( {\gamma \cdot \left( {p_{t} ( \cdot ) - 0.5} \right) + 0.5} \right),1} \right],0} \right]$$

where *γ* = 1, *γ* > 1, and *γ* < 1 indicate risk-neutral, risk-aversive, and risk-seeking behavior, respectively. This generates models **s1w**, **s2w**, **d1w**, and **d2w**. For these models, the expected value of a stimulus *Q*_*t*_(·) is computed as the product of the reward magnitude and *F*(*p*_*t*_(·),*γ*).

Participants were then assumed to choose actions stochastically, according to a sigmoidal probability distribution such that choice probability of stimulus A on trial *t* is given by:

6 $$p_{t} ({\text{A}}) = \frac{1}{{1 + e^{{ - \beta \cdot \left( {Q_{t} \left( {\text{A}} \right) - Q_{t} \left( {\text{B}} \right)} \right)}} }}$$

where *β* is the inverse temperature which adjusts the degree of stochasticity in participants’ choices.

### Modeling fitting and selection

To fit the above models to each participant’s behavior, we employed *maximum *a posteriori (MAP) estimation, a Bayesian-based approach that incorporates prior belief about parameter values to avoid overfitting common in a maximum likelihood approach^33^.

Following Suzuki et al., 2012^28^ that used a similar task as well as computational models, we constrained learning rates α, α_+_, α_-_, α_1_, α_2_ with a Beta (4.6,50) prior distribution, probability weighting parameter γ with a Gamma (1.9,1.0) prior distribution, and the inverse temperature β with a Gamma (2.8,0.05) prior distribution. These prior distributions were obtained by minimizing the squared error of the first, second, and third quartiles of each parameter reported in Suzuki et al., 2012^28^ (see their Table S1). Note that this choice of the prior distribution for learning rates is also in accordance with the results reported by Behrens et al., 2007^27^ (see their Fig. 2e). Following Niv et al., 2012^58^, we also tested another less constrained prior distribution Beta (2,2) for learning rates which, however, did not improve model fits so as to outperform the current best model s1w with the original choice of prior distribution described above. For the parameter κ and μ, we did not impose strong hypothesis about their values and used a uniform distribution Beta (1.0,1.0) as their prior distribution.

For each participant, we estimated the free parameters for each model by maximizing the posterior probability of the data with the MATLAB function *fminsearch*. To avoid local maxima, the initial point for the optimization routine is chosen randomly for 1,000 times and the best result is used as the optimal parameter estimate. Given the optimal estimates of model parameters, the best fitting model is selected by comparing the model evidence computed using the Laplace approximation^33^. The model evidence favors models with greater likelihood while penalizes models with an increasing number of free parameters to be estimated. Thus, a greater value of model evidence indicates better, more parsimonious model fit.

## Supplementary Information

Supplementary Information

## Data Availability

The data and custom MATLAB code that support the findings of this study are available from the corresponding author upon reasonable request.
