# Supplementary material for: Sex difference in the weighting of expected uncertainty under chronic stress
Source: Sci Rep. 2021 Apr 22;11:8700. doi: 10.1038/s41598-021-88155-1 (PMC8062471; doi:10.1038/s41598-021-88155-1)
Supplement: Supplementary file 1 — Supplementary Information [file 41598_2021_88155_MOESM1_ESM.docx]

**Sex difference in the weighting of expected uncertainty under chronic stress**

Huijie Lei, Yasuhiro Mochizuki, Chong Chen, Kosuke Hagiwara, Masako Hirotsu, Toshio Matsubara and Shin Nakagawa

**Supplemental Information**

**S1 Table. Demographic information and participants’ characteristics.**

| Variables  (mean ± SD or ratio) | Low stress | | High stress | | Stress & Sex Two-way ANOVA or Chi-square test |
| --- | --- | --- | --- | --- | --- |
|  | Male (n=16) | Female (n=19) | Male (n=11) | Female (n=19) |  |
| Age (years) | 22.03±1.02 | 21.54±1.70 | 22.19±1.69 | 23.90±4.64 | ns |
| Father education ^a^ | 4.63±0.86 | 4.68±1.38 | 4.60±0.97 | 4.50±1.15 | ns |
| Mother education ^a^ | 4.63±0.50 | 4.16±0.77 | 4.18±0.98 | 4.00±0.78 | ns |
| Family income ^b^ | 4.45±1.37 | 3.87±1.46 | 4.43±1.99 | 3.88±2.00 | ns |
| Living alone (Y/N) | 1/15 | 4/15 | 1/10 | 2/17 | ns |
| Regular social activities (Y/N) | 10/6 | 12/7 | 9/2 | 9/10 | ns |
| Perceived stress (PSS) | 15.50±2.34 | 14.37±3.58 | 22.27±3.20 | 23.68±3.79 | Stress: F_1,61_= 85.405, p =3×10^-13^ |
| Working memory (d_2_) | 2.60±0.38 | 2.61±0.29 | 2.63±0.41 | 2.51±0.43 | ns |

**a**, Parents’ education level was coded as 1 for elementary school level, 2 for junior high school level, 3 for senior high school level, 4 for vocational school level, 5 for undergraduate level, 6 for master’s level, and 7 for doctorate level. **b**, Family income (annual) was coded as 1 for ~2,000,000 JPY (upper bound not included), 2 for 2,000,000~4,000,000 JPY, 3 for 4,000,000~6,000,000 JPY, 4 for 6,000,000~8,000,000 JPY, 5 for 8,000,000~10,000,000 JPY, 6 for 10,000,000~15,000,000 JPY, 7 for 15,000,000~20,000,000 JPY, and 8 for 20,000,000~ JPY. Due to missing data, the sample sizes for the four groups were n=16, 10, 19, 18 for father education, and n=11, 7, 15, 16 for family income, respectively.


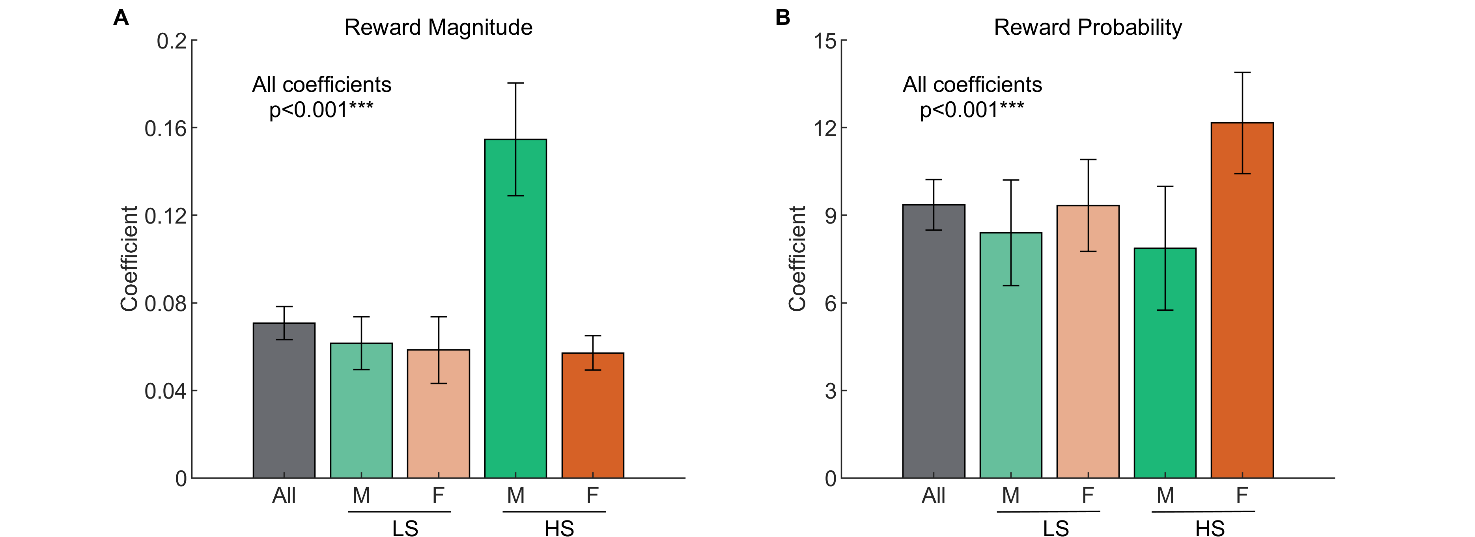


**S2 Fig. Generalized linear mixed model analysis showing the winning model actually captured participants’ behaviors.** Generalized linear mixed model (GLMM) analysis showing the winning model actually captured participants’ behaviors. Participants’ choices on a certain trial were predicted by both the reward magnitude and the reward probability as estimated by the best RL model (i.e., model s1r) on that trial. This was true in all subjects as well as in subjects of each sex-stress group. The GLMM was conducted using the MATLAB R2018b function, *fitglme*. For one option, the GLMM was defined as follows in the Wilkinson notation:

$$Choice\sim1+Reward\_magnitdue+Reward\_probability+\left(1+Reward\_magnitude+Reward\_probability\left|Participant\right.\right)$$

Error bars represent SEM. LS, low stress; HS, high stress; M, male; F, female.

**S3 Fig. Associations between task performance and model estimated parameters across three blocks.** Scatterplot (with regression lines) of the proportion of choosing the option with high expected value as a function of learning rate and probability weighting across three blocks. Only r^2^ and p values that remained significant (p < 0.05) after Bonferroni correction (0.05/12) are shown. The results indicate that higher risk aversion γ but not learning rate α was associated with lower proportion of choosing the option with high expected value in both sexes. Green indicates males, orange indicates females.

**S4 Fig. The sex difference in task performance was not explained by working memory.** There is no effect of sex and stress on working memory (**A**). The sex difference on choosing “correct” choices under high stress remained significant even entering working memory as a covariate (**B**). The coefficients (unstandardized) are obtained using general linear models with sex as the independent variable (male=1, female=0). Error bars represent SEM. * p < 0.05, ** p < 0.01.
